# Uninterrupted monitoring of drug effects in human-induced pluripotent stem cell-derived cardiomyocytes with bioluminescence Ca^2+^ microscopy

**DOI:** 10.1186/s13104-018-3421-7

**Published:** 2018-05-18

**Authors:** Kazushi Suzuki, Takahito Onishi, Chieko Nakada, Shunsuke Takei, Matthew J. Daniels, Masahiro Nakano, Tomoki Matsuda, Takeharu Nagai

**Affiliations:** 1 The Institute of Scientific and Industrial Research, Osaka University, 8-1 Mihogaoka, Ibaraki, Osaka 567-0047 Japan; 2 NIKON CORPORATION, 471, Nagaodai-cho, Sakae-ku, Yokohama, Kanagawa 244-8533 Japan; 3 BHF Centre for Regenerative Medicine, Division of Cardiovascular Medicine, West Wing Level 6, John Radcliffe Hospital, Oxford University, Oxford, OX3 9DU UK

**Keywords:** hiPSC, Cardiomyocytes, Drug screening, Bioluminescence, Ca^2+^, Microscope

## Abstract

**Objective:**

Cardiomyocytes derived from human-induced pluripotent stem cells are a powerful platform for high-throughput drug screening in vitro. However, current modalities for drug testing, such as electrophysiology and fluorescence imaging have inherent drawbacks. To circumvent these problems, we report the development of a bioluminescent Ca^2+^ indicator GmNL(Ca^2+^), and its application in a customized microscope for high-throughput drug screening.

**Results:**

GmNL(Ca^2+^) gives a 140% signal change with Ca^2+^, and can image drug-induced changes of Ca^2+^ dynamics in cultured cells. Since bioluminescence requires application of a chemical substrate, which is consumed over ~ 30 min we made a dedicated microscope with automated drug dispensing inside a light-tight box, to control drug addition. To overcome thermal instability of the luminescent substrate, or small molecule, dual climate control enables distinct temperature settings in the drug reservoir and the biological sample. By combining GmNL(Ca^2+^) with this adaptation, we could image spontaneous Ca^2+^ transients in cultured cardiomyocytes and phenotype their response to well-known drugs without accessing the sample directly. In addition, the bioluminescent strategy demonstrates minimal perturbation of contractile parameters and long-term observation attributable to lack of phototoxicity and photobleaching. Overall, bioluminescence may enable more accurate drug screening in a high-throughput manner.

**Electronic supplementary material:**

The online version of this article (10.1186/s13104-018-3421-7) contains supplementary material, which is available to authorized users.

## Introduction

For decades, a major bottleneck in drug development has been limited availability of patient-derived tissues or cells. This changed with the establishment of human-induced pluripotent stem cells (hiPSC) [1, 2], which can now be differentiated into virtually all cell types in vitro. Patient derived disease-specific hiPSCs recapitulate many disease phenotypes in culture, which may therefore serve as a valuable platform for drug discovery or toxicology testing [3]. This is especially true for the hiPSC-derived cardiomyocytes (hiPSC-CMs) which may enable a paradigm shift in toxicity testing [4]. However thus far they have failed to completely recapitulate established real-world patient based toxicology results in contemporary platforms [5, 6].

Drug-induced changes in hiPSC-CMs can be detected by many methods including classical and automated electrophysiology or established fluorescence imaging modalities [7, 8]. However, the inherent low-throughput of electrophysiological techniques and physiological perturbation arising from phototoxicity of fluorescence excitation may introduce limitations such as recording duration (precluding chronic toxicity studies), or artefacts.

By contrast biocompatibility of bioluminescence emission is robustly demonstrated in many eukaryotic phyla, and as such may provide a better short or long term imaging solution. Bioluminescent proteins (BPs) generate detectable emissions by catalyzing a chemical reaction which consumes a bioluminescent substrate while releasing a photon in the process. This makes them totally independent of external light, reducing phototoxicity. Recent developments of bright BPs such as Nano-lantern, a chimera of Renilla luciferase (Rluc) variant and a fluorescent protein (FP), enable bioluminescence imaging with high signal-to-noise ratio comparable to fluorescence imaging [9, 10]. However, performing bioluminescence imaging at scale is still limited. The faint light associated with bioluminescence requires placement inside an opaque box in order to exclude background light. This factor makes it difficult to test the enormous chemical libraries in a high-throughput manner.

Here we develop a new bioluminescent Ca^2+^ indicator GmNL(Ca^2+^) for hiPSC-CM imaging together with a customized light-tight box which contains a liquid dispenser and regional temperature control and can easily be installed on existing fluorescence microscopy. Combining GmNL(Ca^2+^) with the environmental modifications we demonstrate prolonged visualization of Ca^2+^ transients is improved by bioluminescence compared to fluorescence imaging. Consequently this strategy in hiPSC-CMs may bring value to drug development, particularly in chronic toxicology studies where there is an unmet need.

## Main text

### Materials and methods

#### Gene construction of GmNL(Ca^2+^)

General molecular biology experiments were conducted as described [11]. The sequences of all the oligonucleotides (Hokkaido System Science, Hokkaido, Japan) used in this study are provided in Additional file 1. Each of the cDNAs of C-terminally deleted Gamillus [12] mutants (ΔC8–11) were amplified by PCR and digested with *Bam*HI and *Kpn*I. The digested PCR fragments were cloned in-frame into the *Kpn*I/*Eco*RI sites of Nano-lantern(Ca^2+^)_600/pRSETB (Addgene#51976) for bacterial expression. To express the GmNL(Ca^2+^) in mammalian cells, PCR-amplified GmNL(Ca^2+^) genes were inserted into a pcDNA3 mammalian expression vector using *Bam*HI and *Eco*RI sites.

#### Protein purification and characterization

Recombinant Gamillus-based NL(Ca^2+^)_variant proteins with N-terminal polyhistidine tags were expressed in *E. coli.* (JM109(DE3)), purified, and spectroscopically characterized as described [11].

#### Adeno-associated virus (AAV) production

For the Adeno associated virus expression system, pHelper and pAAV-DJ were obtained from *Cell Biolabs*, *Inc.* (San Diego, CA, USA). The cDNA of GmNL(Ca^2+^) was replaced with ArchT-GFP sequence in pAAV-CAG-ArchT-GFP (Addgene#29777). AAV production followed the manufacturer’s protocol. The titer of AAV vector was determined by fluorescence titration assay. Briefly, HEK293T cells were transduced with a range of dilutions of AAV encoding GmNL(Ca^2+^). 48 h post transduction, the percentage of infected cells were assayed with fluorescence of Gamillus moiety inside GmNL(Ca^2+^) using fluorescence microscopy. To ensure a single infection event per cell, the dilution with less than 40% fluorescent-positive cells was adopted for calculation of titer. The virus titer was approximately 1 × 10^8^ Infectious units (IFU)/ml.

#### Customized bioluminescence microscopy

A bespoke assay environment for bioluminescence imaging was developed for a Ti-E microscope (Nikon Corporation, Tokyo, Japan) equipped with a x10PlanFluor (NA 0.3) objective lens and a stage modified for onstage environmental control (Tokai Hit., Co, Ltd., Shizuoka, Japan). Drug addition and medium exchange was carried out using PROcellcare 5030 and PPPump 2010 MiMEDA enclosed in light-tight box manufactured for this study (Tokai Hit., Co, Ltd.). Fluorescence and bioluminescence images were acquired with an EMCCD camera iXon-3 (Andor Technology, Belfast, Northern Ireland) using the image acquisition software NIS-Elements 4.60 (Nikon Corporation).

#### hiPSC-CM culture and imaging

iCell^®^ Cardiomyocytes (Cellular Dynamics International, Madison, WI, USA) were purchased, and aggregates of hiPSC-CMs were prepared as spheroids. hiPSC-CMs were treated with 10 μl of crude AAV solution (1 × 10^6^ IFU per aggregates of hiPSC-CMs) for 1–5 weeks before observation. The cells were incubated at 37 °C, 5% CO_2_ and culture medium replaced every 3 days. Just before observation hiPSC-CMs were washed with Tyrodes solution (Sigma-Aldrich, St. Louis, MO, USA) and exchanged for 20 μM coelenterazine-h (Wako Pure Chemical Industries, Osaka, Japan) containing Tyrode solution. For Ca^2+^ imaging using Fluo4, 5.0 μM Fluo4-AM (Thermo Fisher Scientific, Waltham, MA, USA) was loaded onto hiPSC-CMs in Tyrode solution supplemented with 1xPowerLoad (provided with Fluo4-AM) for 1 h at 37 °C. Fluo4 imaging was conducted with an FESH0700 IR cut-off filter (Thorlabs, Newton, NJ, USA) and LED505-C-FL (Nikon Corporation) including Ex500/20 excitation filter, DM515 dichroic mirror and EM535/30 emission filter. The images of GmNL(Ca^2+^) were acquired with 50 or 30 ms exposure times for comparison with Fluo4 or drug screening, respectively. For bioluminescence, 8 × 8 binning was applied to increase photon counts for each pixel.

For drug studies, the basal Ca^2+^ transients of hiPSC-CM were recorded, followed by drug incubation for 10 min, and repeated recording of the Ca^2+^ transient. To preserve drug activity, they were initially kept at 4 °C on one side of the chamber and just before use, the temperature was quickly raised to 37 °C to prevent thermal drift. The information of all the drugs used in this study are provided in Additional file 2.

### Results

#### Development and characterization of a bioluminescent Ca^2+^ indicator

**Fig. 1:**
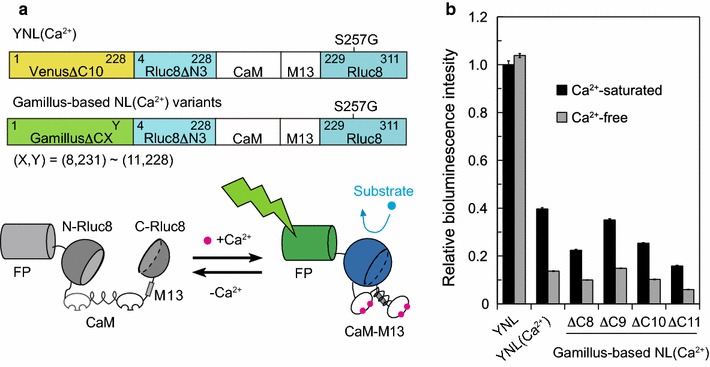
Development of Gamillus based bioluminescent Ca^2+^ indicators. **a** Schematic representation of the domain structures of Gamillus-based NL(Ca^2+^)_variants, **b** relative brightness of recombinant Gamillus-based NL(Ca^2+^)_variants made by deleting 8–11 amino acids at the fusion site between the FRET acceptor and the split luciferase light donor, with or without Ca^2+^. Measurements were performed at least in triplicate, and the averaged data and s.d. are shown

In Nano-lantern, the excited energy produced by an Rluc variant is efficiently transferred to the adjacent FP by FRET. Since the FP has a higher quantum yield (QY) the emitted photon number increases. It was possible to introduce a calcium sensor domain into Nano-lantern to form Nano-lantern(Ca^2+^), but this reduced brightness significantly [9]. To restore brightness we explored fusions to the recently characterized green FP Gamillus [12] which has the highest QY among reported GFPs. We first swapped Venus from Nano-lantern(Ca^2+^) with various C-terminal truncated Gamillus (Δ8–11) constructs to improve FRET efficiency. The resultant fusion proteins are designated as Gamillus-based NL(Ca^2+^)_variants hereafter (Fig. 1a). Of the tested Gamillus-based NL(Ca^2+^)_variants, we found that the Δ9 deletion mutant exhibited a 140% signal change with comparable brightness to YNL(Ca^2+^) when Ca^2+^-bound (Fig. 1b). Ca^2+^ titration revealed that the dissociation constant (*K*_d_) for Ca^2+^ was 240 nM. To compare the performance of these Ca^2+^ indicators to a known standard, we expressed each Gamillus-based NL(Ca^2+^)_variant in HeLa cells. Upon stimulation with histamine, an acute Ca^2+^ spike followed by Ca^2+^ oscillations with smaller amplitudes were detected with sampling rates up to 10 Hz (Additional file 3).

#### Customized bioluminescence microscopy

**Fig. 2:**
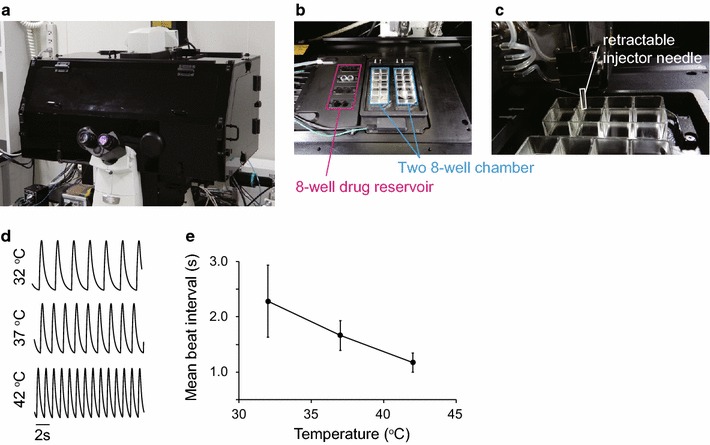
Stage customisation for environmental control, and drug addition during bioluminescence imaging. **a** The observation area was covered with a light-tight box, **b** with stage adjustments to hold two 8-well chamber slides (cyan) and eight drug reservoirs (magenta), **c** the automatic dispenser system inside the light-tight box. **d** time course of Fluo-4 fluorescence intensity in hiPSC-CM at 32, 37, and 42 °C. The measurements were triplicated at each temperature, and representative data are shown. **e** Temperature dependency of peak-to-peak interval of hiPSC-CM. The peak-to-peak interval decreased as temperature was raised from 32 to 42 °C. Data points show the mean ± s.e, n = 3

Figure 2a shows an inverted microscope customized for drug screening using bioluminescence imaging. The system is made of a stage-top incubator, and an automatic dispenser inside a light-tight box, enabling drug preparation, drug addition, and medium exchange (Fig. 2b). This incubator itself has two spaces to hold an 8 well chamber slide for cell observation and eight holes acting as drug reservoirs (Fig. 2c). Temperature can be controlled independently for cell observation (25–50 °C) and drug storage (4–50 °C). Since beating parameters of hiPSC-CMs exhibit thermal dependence, the temperature inside the chamber overall should be constant. To validate the performance of the incubator, Ca^2+^ transients of hiPSC-CMs were recorded by Fluo4, a commonly used fluorescent chemical indicator for Ca^2+^, at 32, 37 and 42 °C. Figure 2d shows a typical time course of spontaneous Ca^2+^ transients at each temperature. The peak-to-peak interval between Ca^2+^ transients, commonly employed as a measure of beat rate, decreased linearly from 2.8 ± 0.29 s at 32 °C to 1.2 ± 0.30 s at 42 °C (Fig. 2e, n = 3).

#### hiPSC-CM imaging

**Fig. 3:**
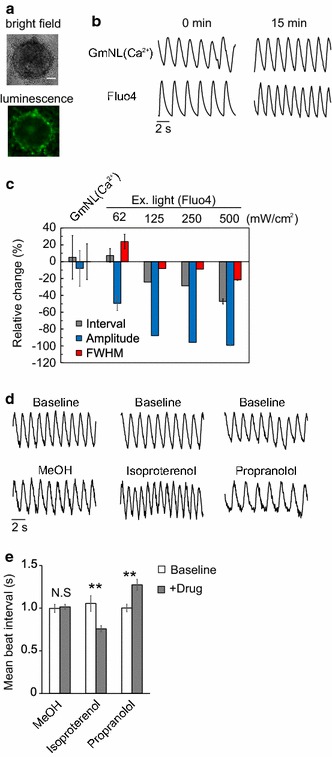
Ca^2+^ imaging with GmNL(Ca^2+^) in hiPSC-CMs. **a** Bioluminescence and bright-field images of hiPSC-CMs expressing GmNL(Ca^2+^). Scale bar, 50 μm. **b** Representative time course of the signal of GmNL(Ca^2+^), and Fluo4 at 0 or 15 min. Images were taken at 20 Hz, 20 μM coelenterazine-h was added just before imaging. Excitation light at 62 mW cm^−2^ for Fluo4 was applied continuously for 15 min. The measurements were replicated (n ≥ 6) for each condition. **c** Relative change in beating parameters after 15 min continuous imaging. Data are presented as mean ± S.D.; n = 6–8. Ca^2+^ imaging with Fluo4 was conducted under four different power densities of excitation light, 62, 125, 250 and 500 mW cm^−2^ respectively. **d** Representative time course of the bioluminescence signal of GmNL(Ca^2+^) before and after treatment with either 1 μM isoproterenol or 40 μM propranolol. The measurements were triplicated for each drug. **e** Mean beat interval before and after treatment; data are presented as mean ± S.D. Two-tailed Student’s t test was performed. **p < 0.01; n = 3

We then assessed Gamillus-based NL(Ca^2+^)Δ9 [hereafter GmNL(Ca^2+^)] signals during spontaneous hiPSC-CM contraction in the customized microscope. GmNL(Ca^2+^) was expressed in cardiomyocyte spheroids by Adeno associated virus infection (Fig. 3a). GmNL(Ca^2+^) was imaged at 20 Hz for 15 min, detecting periodic changes in bioluminescence signal during synchronized contractions (Fig. 3b). The time course of bioluminescence intensity was recorded for the first and last 3 min, and analyzed to estimate three beating parameters; peak interval, peak amplitude, and 50% peak width (FWHM) which approximates to the action potential duration. We compared these results to those obtained using Fluo4 under various illumination power densities (62, 125, 250 and 500 mW cm^−2^ respectively). The amplitude of GmNL(Ca^2+^) did not change significantly during observation (− 8.0 ± 21%, n = 8), in contrast to that of Fluo4 which reduced significantly under all illumination conditions tested (− 50 ± 8.5% at 62 mW cm^−2^ to − 99 ± 0.51% at 500 mW cm^−2^) indicative of dye loss by export or photobleaching (Fig. 3c).

In addition, stability of beating parameters between the start and the end of the observation window was only seen with GmNL(Ca^2+^) (From 1.3 ± 0.36 to 1.3 ± 0.27 s for beat–beat interval, and 0.61 ± 0.14 to 0.59 ± 0.063 s for FWHM, n = 8) and Fluo4 at the lowest power density (62 mW cm^−2^) (from 1.6 ± 0.11 to 1.8 ± 0.10 s for interval, and from 0.48 ± 0.029 to 0.59 ± 0.031 s for FWHM, n = 6). At higher power density reduction in peak-to-peak interval (From 1.9 ± 0.10 to 0.99 ± 0.051 s at 500 mW cm^−2^, n = 7) was seen suggesting extrinsic light illumination can cause significant phototoxicity in this system (Fig. 3c). Collectively, these observations demonstrate that GmNL(Ca^2+^) improves long-term signal stability without physiological perturbations arising from extrinsic illumination. Interestingly the variation between hiPSC-CMs from GmNL(Ca^2+^) was greater than that from cells labelled with Fluo4.

#### Drug-induced changes to the hiPS-CM Ca^2+^ transient

Next we tested whether GmNL(Ca^2+^) can identify expected Ca^2+^ transient changes in hiPSC-CMs induced by well characterized drugs. After addition of isoproterenol, a non-selective β-adrenergic agonist used clinically to increase the heart rate, the mean peak-to-peak interval reduced (from 1.1 ± 0.16 to 0.75 ± 0.070 s, n = 3) (Fig. 3d, e) [13]. Conversely, propranolol, an adrenergic receptor blocker, increased the peak-to-peak interval (from 1.0 ± 0.081 to 1.27 ± 0.11 s, n = 3) as expected (Fig. 3d, e) [14]. Similarly GmNL(Ca^2+^) could correctly elicit the rate related Ca^2+^ transient alterations induced by Dopamine, and Doxazosin (Additional file 4). These results suggested that GmNL(Ca^2+^) is able to report bidirectional drug effects in hiPSC-CMs using a drug dispensing system based inside the on-stage environmental control conditions needed for bioluminescence imaging.

### Discussion

GmNL(Ca^2+^) enables imaging free from the problems of phototoxicity and photobleaching, which plague fluorescence imaging. In contrast to our expectations, GmNL(Ca^2+^) was slightly dimmer than Nano-lantern(Ca^2+^) at saturating Ca^2+^ concentrations. Saturation mutagenesis at the junction between the light donor and the FRET acceptor might improve the performance of GmNL(Ca^2+^). Although the GmNL(Ca^2+^) measurements in hiPSC-CM show stable Ca^2+^ transients between the beginning and the end of the observation window, the variation between hiPSC-CMs appears high in comparison with that from Fluo4. The observed variability might be attributable to heterogeneous infection of AAV or reduced penetration of the luminescent substrate into the spheroid culture model as either may lower bioluminescence signal.

The spontaneous beating characteristics of cardiomyocytes are sensitive to the physical environment, especially temperature, therefore maintenance of sample environment is crucial. In our system we can independently control the temperature of the sample chamber and the drug reservoir, remotely adding the small molecules without perturbation of imaging environment.

Overall, our study presents a bioluminescent Ca^2+^ indicator and a light-tight box equipped with an automatic dispenser that can be controlled remotely. As a proof-of-concept, we demonstrate a minimally harmful, and operator independent Ca^2+^ imaging strategy in hiPSC-CMs with robust testing of drug-induced changes in Ca^2+^ transients at a scale.

## Limitations

As GmNL(Ca^2+^) is intensiometric indicator, the oscillation from GmNL(Ca^2+^) in hiPSC-CMs should include the fraction of motion artefact in addition to Ca^2+^-dependent signal as previously shown [7]. A negative control using Ca^2+^-insensitive probe will give the insightful information about motion artefact.

## Additional files

**Additional file 1.** Oligonucleotides used in this study.

**Additional file 2.** Drugs used in this study.

**Additional file 3.** Characterization of the bioluminescent Ca^2+^ indicators in HeLa. (a) A series of pseudo-coloured ratio images of HeLa cells expressing GmNL(Ca^2+^), following 10 μM histamine stimulation (arrow). Scale bar, 10 μm. (b) Time course of the *B*/*B*_0_ ratio change at an ROI (white box in (a)). Number indicates the time point of each image in (a).

**Additional file 4.** Evaluation of mean beat interval before and after treatment with either 10 μM Dopamine or 10 μM Doxazosin. Two-tailed Student’s t-test was performed. **p < 0.01; Data are presented as mean ± S.D.; n = 3.

## Abbreviations

hiPSC: human-induced pluripotent stem cells
CM: cardiomyocytes
BP: bioluminescent protein
GFP: green fluorescent protein
AAV: adeno-associated virus
QY: quantum yield
FWHM: full width at half maximum
Rluc: Renilla luciferase
FRET: Förster resonance energy transfer
