# Supplementary material for: Uninterrupted monitoring of drug effects in human-induced pluripotent stem cell-derived cardiomyocytes with bioluminescence Ca2+ microscopy
Source: BMC Res Notes. 2018 May 18;11:313. doi: 10.1186/s13104-018-3421-7 (PMC5960208; doi:10.1186/s13104-018-3421-7)
Supplement: Supplementary file 1 — Additional file 1. Oligonucleotides used in this study. [file 13104_2018_3421_MOESM1_ESM.pdf]

| Name of primer | Oligonucleotide sequence (5' to 3') |
|---|---|
| F-BH1-avGFP_1 | TAGGATCCATGGTGAGCAAGGGCGAG |
| R-KpnI-GamillusDel8 | CCACGGTACCCCACAGATTGCC |
| R-KpnI-GamillusDel9 | CCACGGTACCCAGATTGCCGGC |
| R-KpnI-GamillusDel10 | CCACGGTACCATTGCCGGCCA |
| R-KpnI-GamillusDel11 | CCACGGTACCGCCGGCCACGG |
| R-ER1-x-rluc_311 | GACTGAATTCTTACTGCTCGTTCTTCAGCACTC |
