# Supplementary material for: Uninterrupted monitoring of drug effects in human-induced pluripotent stem cell-derived cardiomyocytes with bioluminescence Ca2+ microscopy
Source: BMC Res Notes. 2018 May 18;11:313. doi: 10.1186/s13104-018-3421-7 (PMC5960208; doi:10.1186/s13104-018-3421-7)
Supplement: Supplementary file 2 — Additional file 2. Drugs used in this study. [file 13104_2018_3421_MOESM2_ESM.pdf]

Name	Manufacture	City	State	Country	Quantity
Histamine Dihydrochloride	Wako Pure Chemical Industries, Ltd.	Osaka		Japan	1g
Isoproterenol hydrochloride	Sigma-Aldrich Co. LLC.	St. Louis	MO	U.S.A.	100mg
Dopamine hydrochloride	Sigma-Aldrich Co. LLC.	St. Louis	MO	U.S.A.	5g
Doxazosin mesylate	Wako Pure Chemical Industries, Ltd.	Osaka		Japan	1g
(±)-Propranolol hydrochloride	Sigma-Aldrich Co. LLC.	St. Louis	MO	U.S.A.	1g
