# Supplementary figures and images for: Uninterrupted monitoring of drug effects in human-induced pluripotent stem cell-derived cardiomyocytes with bioluminescence Ca2+ microscopy
Source: BMC Res Notes. 2018 May 18;11:313. doi: 10.1186/s13104-018-3421-7 (PMC5960208; doi:10.1186/s13104-018-3421-7)

a

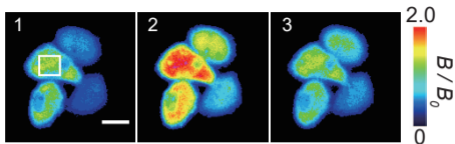

b

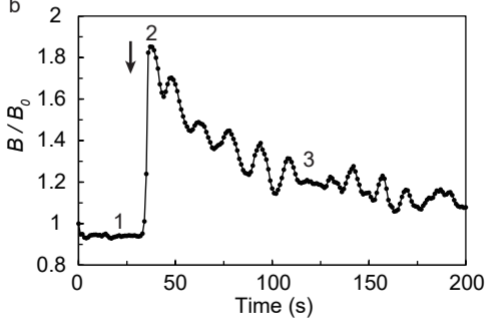

Supplement: Supplementary file 3 — Additional file 3. Characterization of the bioluminescent Ca2+ indicators in HeLa. (a) A series of pseudo-coloured ratio images of HeLa cells expressing GmNL(Ca2+), following 10 μM histamine stimulation (arrow). Scale bar, 10 μm. (b) Time course of the B/B0 ratio change at an ROI (white box in (a)). Number indicates the time point of each image in (a). [file 13104_2018_3421_MOESM3_ESM.pdf]

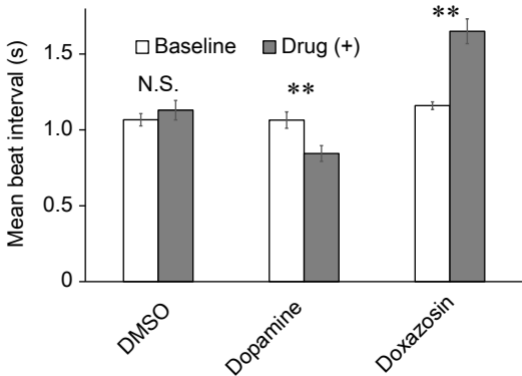

Supplement: Supplementary file 4 — Additional file 4. Evaluation of mean beat interval before and after treatment with either 10 μM Dopamine or 10 μM Doxazosin. Two-tailed Student’s t-test was performed. **p < 0.01; Data are presented as mean ± S.D.; n = 3. [file 13104_2018_3421_MOESM4_ESM.pdf]
